# Adult Onset Acute Flaccid Myelitis: A Case Report

**DOI:** 10.7759/cureus.65294

**Published:** 2024-07-24

**Authors:** Ahmad Peeran, Sameeha Fallatah, Ameera Akour, Ali Alanazi

**Affiliations:** 1 Neurology, King Abdulaziz Medical City Riyadh, Riyadh, SAU; 2 Radiology/Neuroradiology, King Abdulaziz Medical City Riyadh, Riyadh, SAU; 3 Neurology, King Abdullah International Medical Research Center, Riyadh, SAU; 4 Neurology, King Saud bin Abdulaziz University for Health Sciences, Riyadh, SAU

**Keywords:** myelitis, spinal cord, acute flaccid paralysis, afm, acute flaccid myelitis

## Abstract

We report a 23-year-old male who presented with acute dysarthria, dysphagia, and quadriparesis. These symptoms were preceded by fever and headache. His neurological symptoms were progressive and rendered him quadriplegic over three weeks. Extensive workup for infectious, inflammatory, and neoplastic etiologies was negative; however, the clinical course and magnetic resonance imaging of the brain and spinal cord were consistent with the United States Centres for Disease Control and Prevention (CDC) criteria of acute flaccid myelitis. The patient had various lines of therapy, including antimicrobial agents, intravenous immunoglobulin, plasma exchange, and corticosteroids but he was discharged with significant disability. Despite the increasing number of reported cases worldwide, many aspects of this condition remain unknown including its pathophysiology and optimal treatment regimen. In this case report, we shed light on this clinical entity to increase awareness among practitioners worldwide.

## Introduction

Acute flaccid myelitis (AFM) is a clinical syndrome characterized by acute flaccid paralysis associated with spinal cord gray matter lesions. The onset of AFM is usually preceded by symptoms that resemble an upper respiratory tract or gastrointestinal infection. After this, patients develop flaccid limb paralysis, which may progress to involve all limbs and cranial motor nerves, leading to oculomotor dysfunction, facial palsy, and/or pharyngeal dysphagia. Magnetic resonance imaging (MRI) characteristically reveals a longitudinal hyper-intense lesion involving the anterior horn of the spinal cord's gray matter on T-2 sequences. Clinical and laboratory/imaging criteria were introduced by the American Centers for Disease Control and Prevention (CDC) to aid the diagnosis and reporting of these cases [1-3]. Since then, numerous cases have been reported from all over the world, including the United States, Europe, and Japan [1,4,5]. Due to the increasing worldwide prevalence of AFM, the prompt recognition and reporting of patients with this condition is crucial to better characterize the disorder and provide research guidance to help establish effective treatment protocols. In this article, we report the case of a 23-year-old male diagnosed with AFM to bring the attention of physicians working in our region to this clinical entity which will facilitate early diagnosis and avoid unnecessary investigations.

## Case presentation

A 23-year-old, previously healthy Saudi male presented to a regional hospital in Buraydah with a one-week history of progressive neurological symptoms that started with fever, holocephalic headache, and blurred vision. Then, he developed dysarthria, dysphagia, and quadriparesis, which was more prominent on the left side. The systems review was remarkable for abdominal pain that started a few days before the onset of neurological symptoms. He was initially admitted to a hospital in Buraydah for three days. Brain computed tomography (CT) and Magnetic Resonance Imaging (MRI) scans were done and were unremarkable. Two days after his discharge, he presented to our hospital. On the initial examination, the patient was alert and oriented to the person, place, and time. Comprehension was intact but he had dysarthric speech. Vitals were within normal ranges. He had bilateral ophthalmoparesis with bilateral conjugate gaze-evoked nystagmus and bilateral facial weakness. Power examination revealed 2/5 on the left side and 4/5 on the right side, based on the Modified Medical Research Council scale for muscle strength. Deep tendon reflexes were absent in all limbs. The sensory examination was normal. He was admitted, and over the following eight days, the patient's clinical condition deteriorated gradually; he had a fever, developed flaccid quadriplegia, and became mute. On the eighth day, he was transferred to the ICU and intubated.

Initial serum laboratory investigations showed that the complete blood cell count, electrolytes, renal function, and coagulation profile were all within the normal ranges. Cerebrospinal fluid analysis was done several times, and it initially showed lymphocytic pleocytosis, which improved over the course of the disease (Table 1). Infectious causes and autoimmune etiologies were investigated, and all tests were non-revealing (Table 2).

**Table 1:** Cerebrospinal fluid analysis Reference normal parameter range: White cell count: 0-5 x 19^6/L, Red cell count: 0-10 x 10^6/L, Protein: 0.15-0.40 g/L, Glucose: 2.2-3.9 mmol/L

Date	White Cells	Red cells	Protein	Glucose
11/9/2021	53	2	1.04	3.6
14/9/2021	18	3	1.21	4.2
3/10/2021	16	2803	0.47	6.1
29/12/2021	2	2	1.9	3.2
17/1/2022	<1	<1	1.68	3

**Table 2:** Infectious disease and Immunological work up All tests were non-revealing. Ig: Immunoglobulin, PCR: Polymerase chain reaction.

Serum	Respiratory	Cerebrospinal Fluid
Japan Encephalitis virus IgG and IgM	Mycoplasma pneumonia	Human herpes virus 1
Tick born disease (FSME antibodies)	Severe acute respiratory syndrome coronavirus 2 (SARS- COV-2)	Human herpes virus 2
Extractable nuclear antigen antibodies	Bordetella Pertussis	Varicella zoster virus
Epstein-Barr Virus-Early antigen	Bordetella Parapertussis	Human Herpeesvirus-6
Epstein-Barr Virus-|gG	Influenza A	Hyman parechoviruse RY
Epstein-Barr Virus-IGM	Influenza B	Cytomegalovirus
Cytomegalovirus-IgM	Human metapneumovirus	Enterovirus
Cytomegalovirus-IgG	Middle east respiratory syndrome coronavirus	Listeria monocytogenes
West Nile Virus (IgM, IgG)	Respiratory synctial virus	Syphilis - VDRL
Polio virus antibody type 1-3 neutralizing antibody	Human coronavirus NL63	Escherichia Coli K1
Toxoplasmosis Gondi DNA PCR	Human adenovirus	Hemophilus influenza
Human T-Lymphotropic Virus 1 and 2	Parainfluenza virus 1-4	Neisseria Meningitides
Brucella	Rhinovirus/enterovirus	Streptococcus Agalactiae
Syphilis	Human coronavirus 229e	Streptococcus pneumonia
Tuberculosis Polymerase chain reaction (PCR)	Human coronavirus 0C43	Tuberculosis PCR
Quantiferon	Human coronavirus HKU1	Cryptococcus neoformans/gattil
Schistosoma antibody	Chlamydophila pneumonia	
Herpes Simplex Virus 1 and 2 v Anti-Nuclear Antibody		
Anti-Smooth Muscle Antibody		
Anti-Mitochondrial Antibodv		
Anti Myelin oligodendrocyte glycoprotein antibodies		
Anti-aquaporin 4 antibodies		

**Figure 1:**
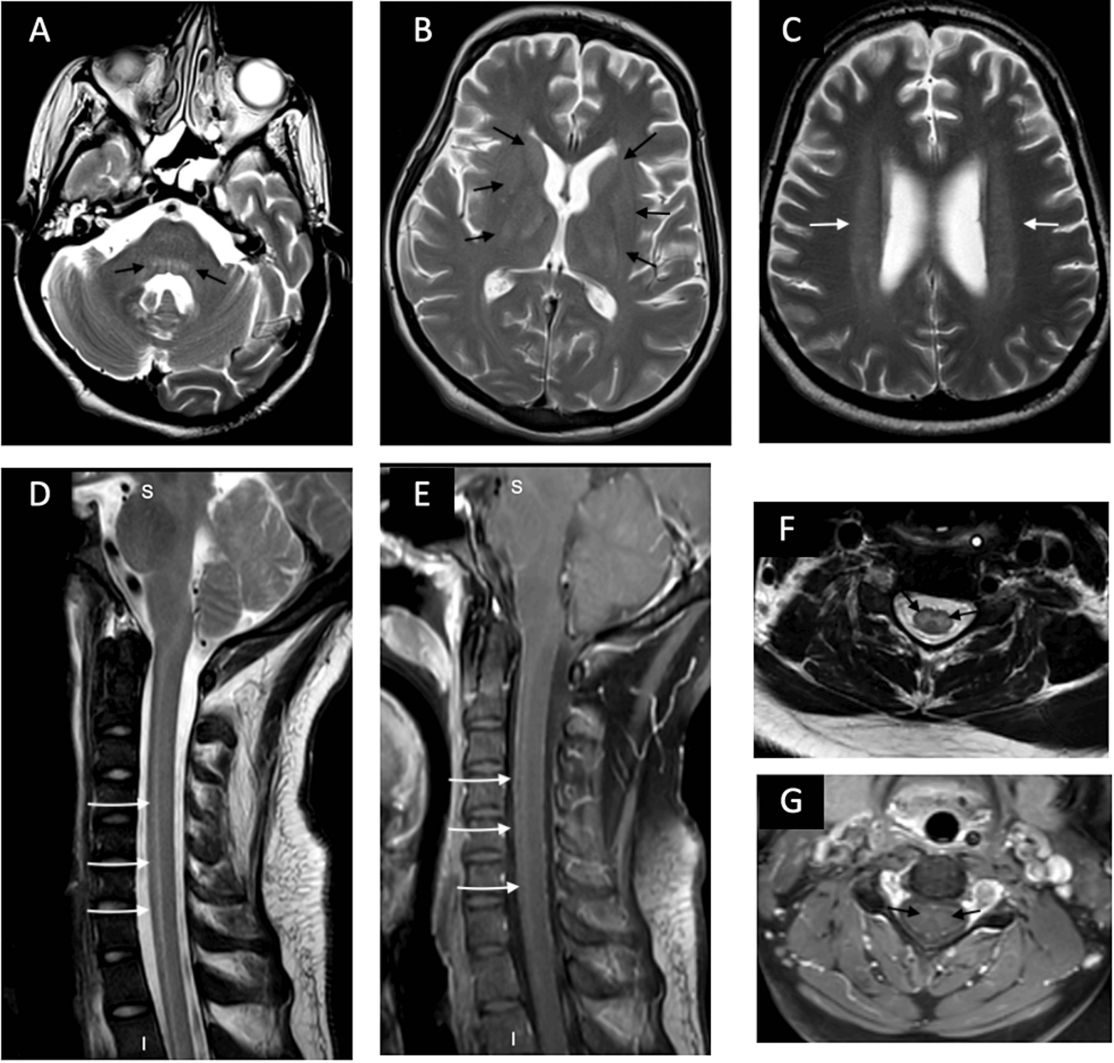
Brain and spinal cord MRI A, B, and C axial T2-weighted images of the head show bilateral symmetrical hyperintensity in the dorsal pons, deep gray matter, and deep white matter (black arrows in A and B, white arrows in C). D and F are sagittal and axial T2-weighted images that show longitudinal hyperintensity mostly involving the central cord, where the gray matter is situated (White arrows in D, black arrows in F). E and G sagittal and axial post-contrast T1-weighted images demonstrate faint contrast enhancement of the same abnormality  (White arrows in E, black arrows in F).

**Figure 2:**
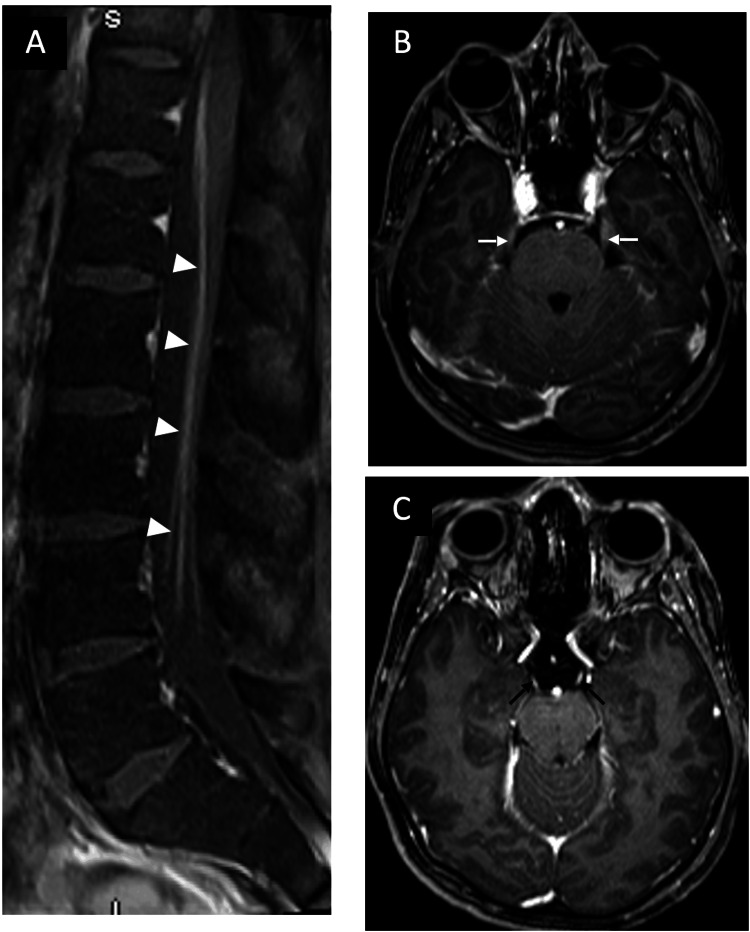
Cranial nerve and nerve root enhancement A, sagittal post-contrast T1-weighted image of the lumbar spine demonstrates anterior cauda equina nerve root enhancement (arrowheads). B and C, axial post-contrast T1-weighted images demonstrate trigeminal (white arrows) and oculomotor (black arrows) nerve enhancement, respectively.

Furthermore, 16s rRNA sequencing for bacteria and fungi was done on the patient's CSF, and it revealed no trace of microbial DNA. Motor nerve conduction study (NCS) showed normal velocity and severely decreased compound muscle action potentials in all limbs. Sensory NCS was normal. Brain CT and MRI were repeated in our center, and they were initially unremarkable. However, spinal cord MRI showed a T2 hyper-intense signal involving the gray matter of the cervical and thoracic spinal cord. Subsequent brain and spine MRI showed basal ganglia and brainstem lesions, along with post-contrast enhancement in the cauda equina (Figures 1, 2). Also, whole body imaging with PET/CT scan was done, and it was negative for signs of neoplastic or autoimmune conditions.

The patient was treated empirically for possible bacterial and viral causes of meningoencephalitis with meningeal doses of ceftriaxone, vancomycin, and acyclovir. However, there was no improvement in his condition and his extensive infectious disease workup was negative. Due to this, treatment for autoimmune conditions was started empirically with intravenous immunoglobulins (IVIG) for five days, but there was no improvement in his condition. One week later, a trial of intravenous methylprednisolone at a dose of 1000 mg was given for three days, after which it was switched to 40 mg for a total duration of 14 days. Also, he was started on plasma exchange but no improvement in his clinical condition was noted. A second trial of steroids was started two months later with methylprednisolone 1000 mg IV for six days, followed by oral prednisone 60 mg.

**Figure 3:**
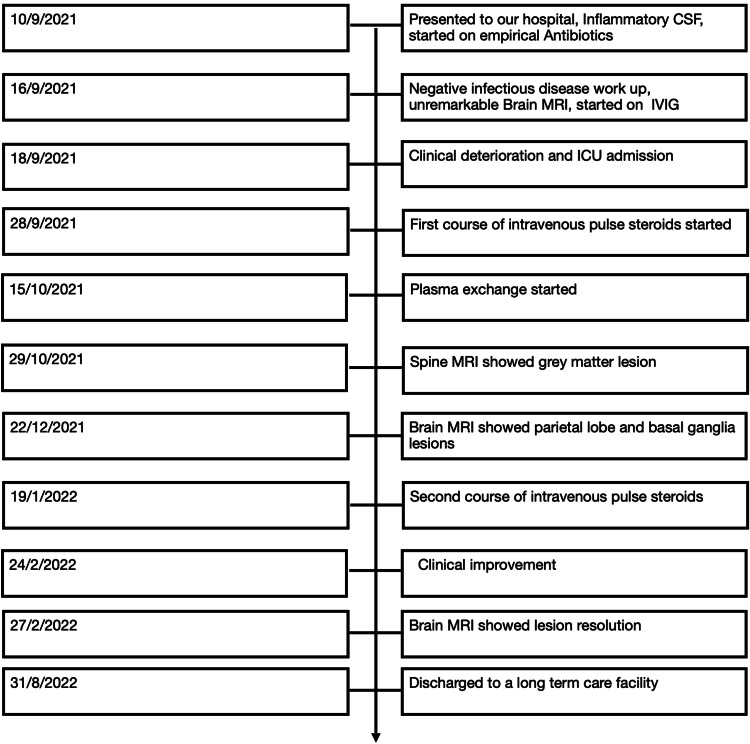
Timeline of patients hospital course

One month after the second course of steroid treatment, he was able to move his tongue in and out of his mouth and to move his head up and down, which made him able to answer simple questions by nodding his head for yes and staying still for no. Upon discharge, the patient was quadriplegic, on percutaneous gastrostomy tube feeding, and requiring mechanical ventilation via tracheostomy. A complete timeline of the patient's hospital course can be reviewed in Figure 3.

## Discussion

The patient presented to our hospital with fever, rapidly progressive flaccid paralysis, and CSF lymphocytic pleocytosis. So, he was started on empirical meningoencephalitis antimicrobial agents. However, the infectious disease workup did not yield any positive results and the patient's clinical condition continued to deteriorate. This raised the possibility of an immune-mediated disease process. Therefore, he received intravenous immunoglobulins (IVIG), plasma exchange, and two courses of pulse steroid therapy. However, his clinical condition did not improve, and the workup for immune-related myelitis, such as anti-aquaporin-4 antibodies and anti-myelin oligodendrocyte glycoprotein (MOG) antibodies, also, showed negative results. Bearing in mind his clinical course, the predominant involvement of the gray matter in the spinal cord, and negative extensive workup, AFM was considered as a diagnosis. 

According to the CDC, AFM is characterized by rapid-onset flaccid weakness associated with distinct abnormalities of the spinal cord gray matter on MR imaging. A confirmed case of AFM should meet both clinical and radiological criteria. It is defined clinically as an acute-onset flaccid weakness of at least one limb that cannot be attributed to any other diagnosis. The radiological criteria stipulate the presence of a spinal cord lesion that mainly affects the gray matter and involves more than one segment. Also, spinal cord lesions due to neoplastic, vascular, or anatomic abnormalities must be excluded [1]. Our patient's clinical picture of acute flaccid paralysis, along with MRI findings of longitudinally extensive myelopathy that involves gray matter, is consistent with the CDC's criteria for a confirmed case of AFM.

AFM, in its current case definition, was first observed in 2012, but it came to public attention in the summer of 2014 after an outbreak of flaccid weakness cases in children in the United States. It was linked to Enterovirus D-68 (EV-D68), as the outbreak coincided with a rise in respiratory infection caused by EV-D68. Also, a mouse-model study showed that neonatal mice developed flaccid paralysis after they were infected by the virus. Nevertheless, the causative relationship has not been confirmed in humans [6-9]. Moreover, other viruses, such as enterovirus 71, West Nile virus, and Japanese encephalitis virus, are known to cause clinical manifestations and radiological features that are similar to AFM, which makes heterogeneity of causes for this clinical syndrome a possibility [3]. In the following years, similar cases were reported from other countries on different continents, including Turkey, Japan, Argentina, and Australia, and many of these cases have been linked to infection with EV-D68 [4,5]. The majority of patients with AFM were children or young adults, so the initial case definition restricted the age to less than 21 years. However, this restriction was removed in 2015 [2]. In addition to the typical presentation of flaccid weakness and gray matter involvement of the spinal cord, patients with AFM may have ophthalmoparesis, facial weakness, and evidence of dorsal pons and basal ganglia involvement in brain MRI [5,10]. The management of AFM is mainly supportive. Intravenous immunoglobulins, steroids, plasma exchange, and antiviral agents were used but without evidence of efficacy. Also, extensive rehabilitation and nerve transfer surgery may be useful in selected cases.

Nonetheless, prognosis is generally poor, and full recovery was reported in less than 10% of cases [2,11,12]. In Saudi Arabia and the other countries in the Arabian Peninsula, there are no reported cases of respiratory infection caused by EV-D68. However, EV-D68 was discovered in the sewage system and archived biological samples of children with respiratory symptoms in Israel [13,14]. In our patient, all the tests for viruses known to cause polio-like syndromes were negative, but many of the tests were sent after the acute phase of the illness had resolved, which decreased the tests' sensitivity.

In this case, we have many unanswered questions. However, answering them is possible only with vigilant reporting of suspicious cases and active surveillance of the disease and these viruses. This will reveal the actual incidence of this disease. Also, it may lead to confirming the disease's causes, which would help in developing disease prevention methods and improving the therapeutic care provided to these patients.

## Conclusions

Acute flaccid myelitis is a rare emerging neurological disorder that leads to significant disability. Although it has been initially reported in the pediatric population, it can affect patients at any age. The disorder's pathogenesis is not fully understood but is possibly related to a viral infection. There is no proven curative treatment for the disease but early rehabilitation may help. In addition, increasing awareness about this entity in the medical community will shorten the diagnostic process and may help develop preventive measures in the future.
